# Benchmarking multiple gene ontology enrichment tools reveals high biological significance, ranking, and stringency heterogeneity among datasets

**DOI:** 10.3389/fbinf.2026.1755664

**Published:** 2026-01-29

**Authors:** Fábio Henrique Schuster de Oliveira, Felipe Acker Gomes, Bruno César Feltes

**Affiliations:** Laboratory of DNA Repair and Aging, Department of Biophysics, Institute of Biosciences, Federal University of Rio Grande do Sul, Porto Alegre, Brazil

**Keywords:** benchmark, bioinformatics, functional enrichment, gene, ontology, overrepresentation analysis

## Abstract

Functional enrichment analysis (FEA) provides biological meaning from lists of differentially expressed genes and proteins obtained through omics experiments. FEA tools can employ numerous statistical methods and rely on different pathway databases. In this sense, Overrepresentation Analysis (ORA) is one of the most popular methods to perform FEA. Gene Ontology (GO) is arguably the most widely used pathway knowledgebase in FEA. Hence, benchmarking the biological accuracy of ORA-based GO enrichment tools is crucial. Nevertheless, benchmark studies in FEA tend to focus excessively on performance-based metrics rather than on the biological information contained in enrichment results. To identify the differences between popular ORA-based GO enrichment tools and provide data that brings insights into the tools’ biological accuracy and, thus, better suits the application of FEA, we tested 12 popular GO enrichment tools (i.e., DAVID, PANTHER, WebGestalt, Enrichr, ShinyGO, limma, topGO, GOstats, clusterProfiler, g:Profiler, ClueGO, and BiNGO) with randomized datasets as negative controls, a target-oriented and a hallmark datasets as positive controls, and an experiment-derived dataset. Gene sets with 500, 200, 100, and 50 genes were built for each dataset to investigate the impact of input sizes. Using the control datasets, we calculated the FPR and accuracy of the tools based on the semantic similarity between the enriched terms and the target ontologies and assessed overlooked, insightful metrics that reflect the biological informativeness of the results, such as the specificity of enriched GO terms and the prioritization of target ontologies. Additionally, we clustered the FEA results based on term semantic similarity, enabling us to directly compare the biological profiles generated by each tool. Despite employing the same method and functional database, the tools’ results diverged significantly. Our findings reveal considerable variation among tools in terms of informativeness and interpretability of results. Some tools demonstrated strong capabilities in prioritizing target pathways, while others struggled, especially as input size increased. Additionally, we observed that the degree to which the enriched ontologies are related to the expected targets varies across tools, with some being more conservative than others. Together, these results provide powerful insights into the performance characteristics of the analyzed GO enrichment tools and yield new, relevant data for benchmarking FEA tools.

## 1 Introduction

Functional enrichment analysis (FEA) is a widely used method that provides additional biological meaning from lists of differentially expressed genes (DEGs) and proteins obtained primarily from high-throughput omics experiments by identifying enriched “functional descriptions” within omics data. FEA tools use the knowledge contained in functional databases, which associate functional categories with gene lists, such as the Gene Ontology (GO) knowledgebase (Ashburner et al., 2000; Consortium et al., 2023), the Kyoto Encyclopedia of Genes and Genomes (KEGG) (Kanehisa and Goto, 2000; Kanehisa et al., 2025), WikiPathways (Agrawal et al., 2024), and Reactome (Milacic et al., 2024). In this scenario, the GO is one of the most widely used resources in the scientific community for providing functional information on genes and gene products.

Currently, a variety of methods that rely on different databases and statistical approaches have been developed to conduct FEA. Most available methods can be classified into four main classes: Overrepresentation Analysis (ORA), Functional Class Scoring (FCS), Pathway-topology-based (PT), and Network-based (NB). Due to their importance, FEA has become embedded in nearly all omics analysis protocols. However, despite employing the same method, different enrichment tools produce different outputs. Due to such inherent heterogeneity, efforts are made to benchmark the performance of distinct enrichment approaches and tools (Tarca et al., 2013; Bayerlová et al., 2015; Lim et al., 2018; Nguyen et al., 2019; Zyla et al., 2019; Geistlinger et al., 2021; Buzzao et al., 2024). Nevertheless, such benchmark studies tend to focus on comparing the statistical methods (i.e., ORA, FCS, PT, and PT), instead of comparing the results profile generated by them (Tarca et al., 2013; Lim et al., 2018; Geistlinger et al., 2021; Buzzao et al., 2024). Moreover, by using only a few tools to represent a whole class (e.g., DAVID for ORA; GSEA for FCS), these studies neglect the differences among software based on the same method (Dong et al., 2016; Nguyen et al., 2019; Zyla et al., 2019). Another commonly overlooked limitation of benchmarks in the case of FEA is that the comparisons tend to rely solely on standard performance metrics, such as FDR, sensitivity, accuracy, and specificity, which fail to accurately illustrate the performance and behavior of FEA tools, as they disregard the biological information that the results provide.

In this study, we evaluated the behavior of 12 commonly used ORA-based tools (Table 1) that utilize the GO resource for FEA. To evaluate the performance of the selected tools, we used an approach focused on the biological meaning derived from the FEA. We conducted enrichment analysis using *random* datasets, a *Hallmark* dataset, a GO Biological Process (*GOBP*) gene set, and a microarray-derived dataset, all split into lists of varying sizes. The random group served as a negative control, while the hallmark dataset was employed as a positive control. Furthermore, the *GOBP* dataset was constructed with predetermined target ontologies to enable the calculation of relevant metrics, including accuracy and FPR, and to assess the tools’ ranking abilities of the target pathways. Finally, we constructed the *Contextual* lists using real high-throughput experiment data to evaluate the differences in the results of various tools in a realistic research scenario. We also used GO term annotation size and depth in the ontology as measures for biological specificity. Such an approach has been used in previous studies, but is not commonly employed in FEA benchmarks (Lewin and Grieve, 2006; Louie et al., 2010; Tomczak et al., 2018).

**TABLE 1:** Selected tools for the comparative analysis and relevant information. The GO version column corresponds to the GO version used at the time of the analyses according to each tool’s documentation. The column “Raw p-value” indicates whether the tool also provides raw p-values alongside their corrected values.

Software	GO version	Custom annotation and GO files	Raw p-value	Platform	Release	Version/Last updated	Reference
DAVID	2025^a^	No/No	Yes	Web	2003	4/1/2024 (DAVID Knowledgebase v2024q1)	Huang et al., 2009; Sherman et al., 2022
PANTHER	2025-02-06	No/No	Yes	Web	2000	v18.0 - 17/09/2023	Mi et al. (2019)
Enrichr	2025^b^	No/No	Yes	Web	2013	8/7/2023	Chen et al. (2013)
WebGestalt	2024^b^	No/No	Yes	Web/R package	2005	2024	Elizarraras et al. (2024)
g:Profiler	2024^b^	Yes/No	No	Web/R package	2007	e112_eg59_p19_25aa4782 - 2025	Kolberg et al. (2023)
ShinyGO	2022^b^	No/No	No	Web	2018	v0.82 - 2/2025	Ge et al. (2020)
ClueGO	2025-03-16	Yes/Yes	Yes	Cytoscape	2009	v2.5.10 - 2023	Bindea et al. (2009)
BiNGO	2013^b^	Yes/Yes	Yes	Cytoscape	2005	v3.0.5 - 2021	Maere et al. (2005)
topGO	2024-09-20	Yes/No	Yes	R package	2006	v2.58.0 - 2024	Alexa A (2024)
clusterProfiler	2024-09-20	Yes/No	Yes	R package	2012	v4.14.6 - 2025	Xu et al. (2024)
GOstats	2024-09-20	No/No	Yes	R package	2006	v2.72.0 - 2025	Falcon and Gentleman (2007)
goana (limma)	2024-09-20	No/No	Yes	R package	2015	Limma v3.62.2 - 01/2025	Ritchie et al. (2015)

Table legends: ^a^ updated daily; ^b^ only the update year is mentioned.

In addition to providing a comprehensive overview of how leading ORA-based tools perform in terms of output consistency and biological relevance, our work also proposes a novel benchmarking strategy to guide the evaluation of future tools in the field. The goal of this work is not to analyze the performance of the ORA tools, but to examine the coherence and profile of the biological information they provide across different inputs, list sizes, and their precision in ranking and identifying expected results.

## 2 Materials and methods

### 2.1 Dataset generation

For each dataset, we created lists with 500, 200, 100, and 50 genes. Furthermore, Entrez ID gene lists were created for all lists by converting their gene symbols using the biomaRt library in R (Durinck et al., 2005; Durinck et al., 2009). Both lists, with gene symbols and Entrez IDs, were used because some tools either accepted or displayed imprecise results for one of the identifiers. Our datasets can be acquired from [https://github.com/LARA-Lab-Aging].

The random lists were generated by selecting 500, 200, 100, and 50 protein-coding *Homo sapiens* genes at random. This process was repeated 5 times to produce 5 different lists for each size.

The *Hallmark* lists were built from the gene sets available in the MSigDB human hallmarks collection (Subramanian et al., 2005; Liberzon et al., 2011; 2015). To create the 500 genes *Hallmark* list, we combined the HALLMARK HYPOXIA, HALLMARK DNA REPAIR, and HALLMARK UV RESPONSE UP gene sets. The 200-gene list contained only genes in the hypoxia set. The 100 and 50-gene lists were built by downsampling the 200-hypoxia dataset.

The *GOBP* lists were generated by combining gene sets from different GO Biological Process datasets in the MSigDB Ontologies collection. To build the lists, we aimed to select ontologies with minimal overlap. The ontology gene sets that compose each gene list are gathered in Supplementary Table S1. Additionally, all lists had their duplicates removed.

The *Contextual* dataset was obtained from the lung cancer GSE18842 dataset available in the Gene Expression Omnibus (GEO) [https://www.ncbi.nlm.nih.gov/geo/], which comprises 46 cancer and 45 control samples (Sanchez-Palencia et al., 2011). Firstly, the dataset was imported into Gene Expression Analysis Platform (GEAP) software, which employs underlying R-based tools through a graphical user interface to perform microarray data analyses (Nunes et al., 2022). Quality analysis was conducted to filter out low-quality data within GEAP, which utilizes the *arrayQualityMetrics* package internally. Samples that failed the quality metrics of at least two of the three metrics analyzed by *arrayQualityMetrics* would be discarded before the Differential Gene Expression (DGE) analysis; however, no samples had to be discarded. Next, DGE analysis was conducted through the “Comparison Between Two Groups” tab in GEAP, which employs the limma package. The parameters used were the default eBayes method and the False Discovery Rate (FDR) correction method (Benjamini and Hochberg, 1995). Finally, the results were filtered for logFC >1 and p-value <0.05, yielding a table with 3,222 DEG (overexpressed = 1,386; underexpressed = 1836). The top 500 DEGs were selected to build the 500 contextual gene list. The same downsampling process as described before was used to create the smaller lists.

### 2.2 Tool selection and FEA

Tools were selected based on the following criteria: (i) tools widely used in the scientific community (≥500 citations on Google Scholar), (ii) tools that have been updated in the past 5 years or allow the usage of up-to-date GO annotations and ontology files, and (iii) tools that are functioning as described in their available documentation. For the FEA, we attempted to maintain the parameters as similar as possible and used the default options when no equivalents were available. All analyses were conducted using human GO annotations. The whole human genome was used as background for the *Random*, *Hallmark*, and *GOBP* sets, whereas the Affymetrix table from GSE18842 served as background for the *Contextual* dataset. In general, either the FDR or the Benjamini-Hochberg (BH) correction method was used, and all ontologies with fewer than 2 genes were removed from the results. Enrichment results for the *Hallmark*, *Contextual*, and *GOBP* datasets are compiled in Supplementary Table S2. All raw outputs are available at [https://github.com/LARA-Lab-Aging]. Additionally, gene-mapping success rates are provided in Supplementary Table S5.

#### 2.2.1 BiNGO

Analyses that employed BiNGO were used to assess overrepresentation and employed the hypergeometric test to compute p-values (Maere et al., 2005). Additionally, the significance level was set to 1, all categories were selected, and the ontology file used was GO_Biological_Process in BiNGO.

#### 2.2.2 ClueGO

ClueGO enrichment results were obtained through the One-sided hypergeometric test (enrichment) with the GO Biological Process annotation set, the evidence option set to “All”, minimum number of genes per ontology set to two, and the BH option for p-value correction (Bindea et al., 2009). Other options available were all unselected.

#### 2.2.3 DAVID

Results from DAVID were obtained by querying the 16 gene lists (Entrez IDs) in the functional annotation tab and selecting the GOTERM_BP_DIRECT chart (Huang et al., 2009; Sherman et al., 2022). The parameters used were EASE ≤1, Count ≥2, and the maximum number of records was set at 10,000. The correction method employed was FDR.

#### 2.2.4 Enrichr

The results in Enrichr were obtained by querying the 16 Gene Symbol gene lists and selecting the GO Biological Process 2025 in the Ontologies tab (Chen et al., 2013). The correction method was BH.

#### 2.2.5 GOstats

Analyses in the GOstatsR package were conducted through the hyperGTest function (annotation = “org.Hs.eg.db”, ontology = “BP”, pvalueCutoff = 1, testDirection = “over”) that is based on the hypergeometric distribution statistical test (Falcon and Gentleman, 2007).

#### 2.2.6 PANTHER

Analyses in PANTHER were conducted with the Statistical Overrepresentation test and the Biological Process complete annotation set (Mi et al., 2019). The statistical test used was Fisher’s exact test, and the correction method employed was FDR. PANTHER could not map Entrez IDs properly. Thus, lists were queried with the Gene Symbols.

#### 2.2.7 ShinyGO

ShinyGO analyses results were generated in the Enrichment tool with the Pathway database option set to GO Biological Process, FDR cutoff to 1.0, and pathway minimal size to 2 (Ge et al., 2020). The redundancy removal option was unselected. Only the top 1,000 enriched ontologies were selected, which is the maximum number of results ShinyGO outputs.

#### 2.2.8 WebGestalt

Analyses in WebGestalt were conducted by using the ORA method option with the Biological Process GO Functional Database (Elizarraras et al., 2024). No redundancy removal method was selected, and the FDR was used as the p-value adjustment method. The top 10,000 ontologies were selected as the results.

#### 2.2.9 clusterProfiler

Enrichment results in clusterProfiler were obtained through the enrichGO function (OrgDB = “org.Hs.eg.db”, ont = “BP”, pAdjustMethod = “fdr”, pvalueCutoff = 1, minGSSize = 2), which uses the hypergeometric distribution to conduct statistical analysis (Xu et al., 2024).

#### 2.2.10 g:Profiler

The analyses with g:Profiler were conducted in the R package gprofiler2 through the gost function (organism = “hsapiens”, significant = F, user_threshold = 1, correction_method = “fdr”, sources = “GO:BP”) (Kolberg et al., 2023).

#### 2.2.11 goana (limma)

The goana function from the limma R package was used to conduct the analyses, with the FDR parameter set to 0.05 and the species set to “Hs” (Ritchie et al., 2015). The custom background option was used with the Contextual lists.

#### 2.2.12 topGO

Analyses with topGO were conducted with the tool’s default weight01 algorithm, Fisher’s exact statistical test, org. Hs.eg.db annotation data, and the BP ontology set (Alexa, 2024). Subsequently, p-value correction with FDR through R’s p. adjust function before removal of ontologies annotated to less than 2 genes in the input list to ensure adequate p-value correction.

### 2.3 GO term specificity assessment

GO term biological specificity was assessed based on the term’s annotation size and its depth in the ontology structure. Both properties have been widely employed to root the evaluation of term specificity (Lewin and Grieve, 2006; Louie et al., 2010; Tomczak et al., 2018). Additionally, the link between them and biological specificity is highly intuitive: a term tends to be more general as more genes are associated with it, and, because the ontology is hierarchical, terms deeper in the hierarchy tend to be more biologically precise. Metrics were retrieved using the GOATOOLS Python library (Klopfenstein et al., 2018).

### 2.4 Semantic similarity analysis

Semantic similarity (SS) analyses were conducted using GOATOOLS, which implements multiple methods to determine semantic similarity between two GO terms. We selected Wang’s method (Wang et al., 2007) to conduct the SS analyses, as it relies solely on the GO Directed Acyclic Graph structure to define SS and attempts to translate the similarity of two ontologies into biological meaning. The GO’s relationships ‘is_a’ and ‘part_of’ with edge scores of 0.8 and 0.6, respectively, were used to determine Wang’s SS score.

### 2.5 Metrics calculation

To calculate the defined metrics, we used the enrichment results obtained with the *GOBP* dataset. True positives (TP) were defined as statistically significant ontologies (adjusted p-value <0.05) that had a Wang’s semantic similarity (SS) score of at least 0.7 with at least one of the target ontologies for that input list. Conversely, false negatives (FN) were ontologies with a Wang’s SS score of at least 0.7, but that were not statistically significant. Similarly, false positives (FP) and true negatives (TN) were ontologies with low maximum semantic similarity (Wang’s SS score <0.3) compared to the original target pathways, which were statistically significant or not, respectively. These Wang’s SS score thresholds were selected to center the analysis on the ontologies that are very similar (Wang’s SS score >0.7) to one of the targets and are, therefore, expected in the results; and on ontologies that are very dissimilar (Wang’s SS score), thus are unexpected.

### 2.6 Enriched GO terms network construction and clustering

To group the enriched ontologies into functionally similar clusters, we built interaction networks for all FEA results, in which the edge score between two GO terms was their respective Wang’s SS scores. The networks were then pruned with an edge-weight cutoff of 0.5 to remove edges connecting dissimilar ontologies. The resulting ontology similarity networks were clustered using Markov Clustering (MCL) (Van Dongen, 2008), one of the most robust algorithms for clustering biological data (Brohée and van Helden, 2006; Satuluri et al., 2010; Lim et al., 2019). Of the four Markov Clustering inflation values tested – 1.5, 2.0, 3.5, and 5.0 –, the 5.0 value yielded GO clusters with the highest biological accuracy. Finally, we classified the 15 largest clusters that contained 3 or more ontologies for all the clustered FEA results (Supplementary Tables S3, S4).

**FIGURE 1:**
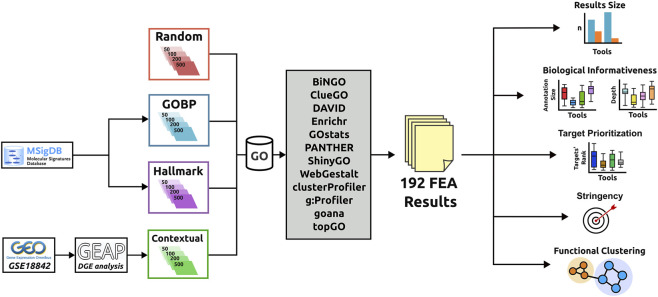
Procedure developed to evaluate the 12 ORA-based GO enrichment tools. Four different datasets were generated, each containing inputs with 500, 200, 100, and 50 genes. FEA results were then analyzed comparatively, and tools’ performance was assessed based on (i) the number of enriched ontologies, (ii) the biological informativeness of their results, (iii) target prioritization, (iv) stringency levels, and (v) biological profile.

Our pipeline is summarized in Figure 1.

## 3 Results

### 3.1 Some tools identify statistically significant ontologies in random datasets

To evaluate how tools handle data with seemingly no biological context, we conducted FEA using multiple randomized lists of human protein-coding genes and analyzed the distributions of the number of enriched GO terms across input sizes. In general, when using the nominal p-value to determine significance, tools do yield enrichment results, and the number of enriched ontologies varies considerably across tools (Supplementary Figure S1).

**FIGURE 2:**
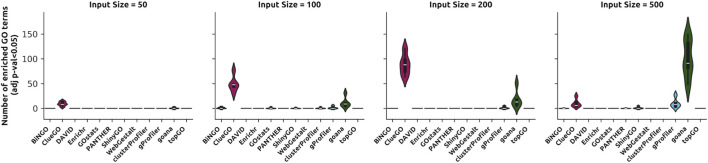
Distribution of the number of enriched GO terms for the input lists in the *Random* dataset. ClueGO and goana tend to yield enrichment results for data with no biological context (i.e., false positives).

Nevertheless, after adjusting for multiple comparisons, most results for all tested tools are not statistically significant (p-value <0.05) (Figure 2), reinforcing the importance of analyzing enrichment results using corrected p-values, as this dramatically reduces the number of false positives, especially when employing ORA (Hung et al., 2012; Wijesooriya et al., 2022). However, there are tools able to retrieve enriched GO terms despite the nature of the dataset and the statistical correction of p-values (Figure 2). Remarkably, ClueGO and goana consistently yielded the largest number of enriched categories for this dataset (Figure 2). In particular, ClueGO displayed a rather interesting behavior: there are substantially fewer enriched GO terms for the 500-lists in comparison to the other input sizes, despite it retrieving larger results when considering nominal p-values (Supplementary Figure S1). Such behavior could reflect how the p-value correction method is implemented and how detected but not enriched GO terms are handled. Ultimately, this suggests that both ClueGO and goana are susceptible to retrieving enriched GO terms that are either irrelevant or insufficiently descriptive.

These results reiterate that, while ORA-based methods can produce significant noise, this can be mitigated by appropriately correcting p-values. Likewise, it reinforces the need to test the coherence and filtering capabilities of biological information provided by ORA-based tools.

### 3.2 The number of enriched ontologies varies greatly among different tools

**FIGURE 3:**
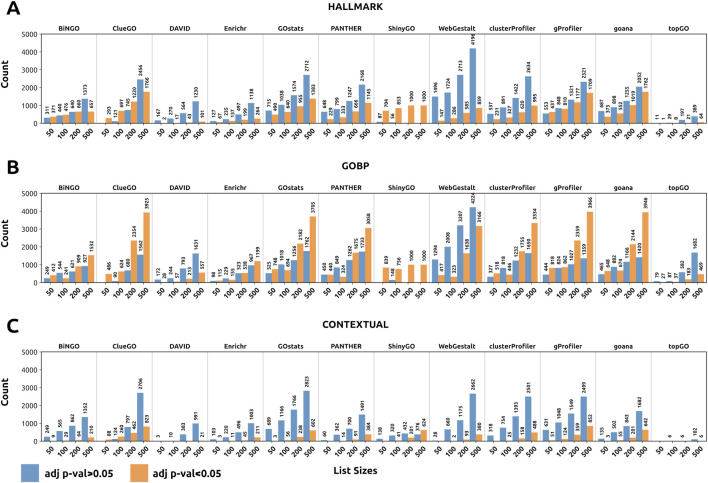
Number of enriched ontologies per tool for each input size in the *Hallmark*, *GOBP*, and *Contextual* datasets. In orange, the number of enriched ontologies (adjusted p-value <0.05). In blue, the number of non-enriched ontologies (adjusted p-value >0.05). **(A)** Number of enriched ontologies for the Hallmark datasets. **(B)** Number of enriched ontologies in the GOBP dataset. **(C)** Number of enriched ontologies in the Contextual datasets. The size of the FEA results varies greatly across tools.

One of the main differences we observed when comparing results across enrichment tools was that the number of enriched GO terms varied significantly in the *Hallmark*, *GOBP*, and *Contextual* datasets (Figure 3). DAVID and topGO were the most conservative tools, retrieving considerably fewer enriched categories than the other tools throughout the three datasets. This could be a double-edged sword: while it identifies fewer enriched categories, potentially highlighting the ontologies closely related to the input, it may also overlook unexpected ontologies that could contribute important biological novelty.

In contrast, ClueGO, GOstats, PANTHER, ShinyGO, WebGestalt, clusterProfiler, g:Profiler, and goana yielded the largest results across the analyzed datasets. In this case, identifying too many enriched GO terms may be detrimental, as they are more prone to false positives and allow the user to choose from an excessively broad range of statistically significant terms, thereby biasing the interpretation of the results toward what is relevant to them. We argue that in such cases, a more stringent approach should be taken to analyze the enrichment results, such as using smaller p-value cutoffs and filtering by term annotation size and overlapping genes.

These results indicate that not all tools may be suitable for all potential research questions, as they retrieve highly heterogeneous sets of ontologies. In this sense, conservative tools might not yield biological novelty, as they are more likely to show expected results. In contrast, tools that retrieve thousands of GOs will not only eclipse relevant and expected results but also force researchers to browse overly extensive lists in search of useful findings.

### 3.3 The level of biological informativeness of FEA varies across tools

To estimate the biological informativeness of the FEA results provided by each tool, we used the median and average of both the annotation sizes and depths of the enriched GO terms as measures of biological specificity (Tables 2, 3). We chose to limit our analysis to the top 20 and top 100 enriched ontologies, arranged by p-values in ascending order, to lessen the impact of greatly varying result sizes and to examine the tools’ ranking preferences. Additionally, since the final step of FEA inherently involves manual interpretation, it is reasonable to consider these portions of the results the most relevant. Both selected metrics were analyzed for the top 20 and top 100 ranked enriched ontologies for all FEA results with the *Hallmark* and *Contextual* datasets.

**TABLE 2:** Table compiling the median annotation size (number of genes associated with a pathway) and the median depth (maximal level in the ontology) of the top 20 and top 100 enriched ontologies (adj P-val ≤0.05) for input lists containing 50 and 500 genes for the HALLMARK dataset.

Tools	List size	HALLMARK							
		Median annotation size		Average annotation size		Median depth		Average depth	
		top20	top100	top20	top100	top20	top100	top20	top100
BiNGO	50	242.50	172.00	937.85	727.77	4.00	4.00	4.00	3.84
	100	200.50	218.00	425.65	751.54	4.50	4.00	4.35	3.65
	200	152.00	269.00	336.30	839.74	4.50	4.00	4.25	3.79
	500	1417.50	380.50	2088.15	903.71	3.00	4.00	3.35	3.89
ClueGO	50	176.00	122.00	206.50	141.09	5.00	6.00	5.05	6.42
	100	143.00	208.00	202.85	340.03	8.00	6.00	7.60	6.29
	200	143.00	310.00	187.85	765.81	8.00	5.00	7.85	5.84
	500	597.50	321.00	2385.75	1269.15	5.50	5.00	5.70	5.89
DAVID	50	55.50	46.50	163.90	112.38	5.00	5.50	6.15	5.72
	100	49.00	48.50	100.65	111.19	6.00	6.00	6.65	5.98
	200	49.00	51.00	153.25	125.29	6.00	6.00	6.95	5.96
	500	82.00	49.50	177.90	118.44	7.50	7.00	7.70	6.77
ENRICHR	50	67.00	56.50	213.60	168.55	6.00	6.00	5.85	6.58
	100	59.50	44.50	158.60	134.59	6.00	6.00	6.20	6.93
	200	31.00	49.00	37.15	182.12	9.00	6.00	9.00	6.73
	500	54.00	49.00	104.55	140.33	8.00	7.00	7.90	7.47
GOstats	50	560.00	341.50	744.50	1116.91	4.00	4.00	3.85	5.09
	100	121.00	217.00	184.10	616.74	8.00	6.00	7.45	6.17
	200	116.00	256.50	156.05	650.91	8.00	5.50	7.95	6.11
	500	406.00	337.00	1816.60	1368.79	7.00	5.00	6.40	5.87
PANTHER	50	320.00	234.00	687.70	945.94	4.00	4.00	4.40	5.33
	100	69.00	170.50	98.90	735.85	8.00	5.50	7.95	6.01
	200	69.00	250.50	98.90	1080.93	8.00	5.00	7.95	5.85
	500	515.50	399.00	3114.90	1607.08	5.50	5.00	5.60	5.63
ShinyGO	50	749.50	644.50	1267.30	1128.00	3.50	4.00	3.70	4.17
	100	297.00	672.00	743.00	1054.52	4.00	4.00	4.45	4.42
	200	156.00	637.50	245.20	1114.78	5.00	4.00	5.45	4.61
	500	1172.50	652.50	2094.35	1319.82	3.00	4.00	3.65	4.59
WebGestalt	50	324.50	206.50	516.10	424.10	4.00	5.00	4.20	5.67
	100	115.50	197.00	172.90	476.56	8.00	5.50	7.45	6.05
	200	111.00	247.50	152.80	521.66	8.00	5.50	7.70	5.90
	500	348.50	244.00	373.60	495.86	7.00	6.00	6.85	6.10
clusterProfiler	50	191.50	145.00	200.95	170.61	5.00	5.50	5.10	6.18
	100	117.00	129.00	155.30	161.49	8.00	6.00	7.65	6.48
	200	117.00	156.00	137.60	179.15	8.00	6.00	8.00	6.64
	500	159.00	162.50	206.55	183.64	8.00	7.00	7.45	7.04
gProfiler	50	813.50	552.50	1692.80	1087.44	3.50	4.00	3.45	4.96
	100	126.50	263.50	250.50	893.26	7.50	5.00	7.30	5.70
	200	113.00	255.50	172.95	743.64	8.00	5.00	7.50	5.98
	500	533.50	466.50	1344.90	1437.14	5.00	5.00	5.30	5.51
goana	50	2688.50	1268.50	5214.05	2832.59	3.00	3.00	2.90	3.79
	100	369.00	633.50	3386.25	2186.26	4.50	4.00	5.45	4.94
	200	122.00	411.50	2454.80	1978.42	7.50	4.50	6.65	5.42
	500	6075.50	1674.50	6540.25	3172.27	3.00	4.00	3.10	4.74
topGO	50	22.50	190.50	247.35	352.82	6.00	5.00	5.90	5.19
	100	27.50	107.50	61.95	226.10	6.00	6.00	6.70	5.68
	200	64.00	47.50	119.70	175.35	6.00	6.00	6.80	5.97
	500	86.00	45.00	308.95	154.66	7.00	7.00	7.40	7.35

**TABLE 3:** Table compiling the median annotation size (number of genes associated with a pathway) and the median depth (maximal level in the ontology) of the top 20 and top 100 enriched ontologies (adj P-val ≤0.05) for input lists containing 50 and 500 genes for the CONTEXTUAL dataset.

Tools	List size	CONTEXTUAL							
		Median annotation size		Average annotation size		Median depth		Average depth	
		top20	top100	top20	top100	top20	top100	top20	top100
BiNGO	50	362.00	238.00	1033.75	518.78	3.00	4.00	3.00	3.77
	100	407.50	159.50	1081.00	439.40	3.50	4.00	4.05	4.50
	200	435.00	165.50	901.35	439.74	3.00	4.00	3.10	4.42
	500	503.50	156.50	1147.35	505.01	3.00	4.00	2.90	4.21
ClueGO	50	81.50	82.00	89.55	84.78	5.50	6.00	5.45	6.12
	100	41.00	70.50	171.70	128.64	5.50	5.50	5.85	5.81
	200	452.00	287.00	726.60	502.23	3.50	5.00	4.15	5.27
	500	386.00	413.50	1238.75	1237.82	4.00	4.00	4.30	4.80
DAVID	50	52.50	121.00	142.85	267.95	5.50	6.00	5.40	5.82
	100	57.50	48.50	118.50	109.42	5.00	5.50	5.20	5.83
	200	62.00	52.50	126.00	136.57	5.50	6.00	5.55	5.85
	500	63.00	48.00	103.90	98.56	6.00	6.00	5.75	5.59
ENRICHR	50	53.50	107.50	100.60	191.32	6.00	6.00	5.95	6.14
	100	29.00	44.00	62.75	100.97	6.00	6.00	6.20	6.59
	200	50.50	35.50	121.25	125.01	5.50	6.00	6.35	6.61
	500	74.00	54.50	123.65	137.87	5.50	6.00	6.25	6.40
GOstats	50	815.50	266.50	1351.05	985.37	3.00	4.00	3.35	4.64
	100	511.50	350.50	1493.20	1048.72	4.50	4.00	4.05	4.63
	200	324.00	323.50	1145.40	932.89	4.00	4.00	4.15	4.83
	500	371.50	388.00	1176.10	1106.70	4.00	4.00	4.35	4.86
PANTHER	50	629.00	172.00	1395.40	1044.67	3.50	5.00	3.80	4.86
	100	287.00	270.50	1404.80	845.76	5.00	5.00	4.90	4.97
	200	843.00	271.00	1631.10	974.72	3.00	5.00	3.25	5.19
	500	352.00	320.00	940.85	1369.69	4.00	4.50	4.05	5.08
ShinyGO	50	638.50	334.00	842.30	838.00	4.00	5.00	4.40	4.67
	100	357.50	334.00	821.70	746.90	5.00	4.00	4.50	4.70
	200	377.00	426.00	844.45	780.82	4.00	5.00	4.15	5.19
	500	355.50	378.50	684.85	882.43	4.50	4.00	4.95	4.92
WebGestalt	50	562.00	189.00	603.80	485.86	4.00	5.00	3.90	5.15
	100	318.00	296.50	657.95	481.40	4.50	5.00	4.20	4.84
	200	318.00	277.50	644.05	476.15	4.00	4.00	4.35	4.95
	500	317.50	306.50	568.90	496.28	5.00	5.00	4.95	5.21
clusterProfiler	50	114.00	100.00	154.05	155.87	5.00	6.00	5.60	6.08
	100	174.50	67.50	187.25	137.12	5.00	6.00	5.50	5.73
	200	170.00	96.50	211.65	153.25	5.50	6.00	5.60	6.10
	500	170.00	135.50	216.35	180.17	6.00	6.00	6.05	6.07
gProfiler	50	1321.00	623.00	3375.20	1966.80	3.00	4.00	2.70	4.05
	100	1344.50	697.50	2385.50	1520.07	3.00	4.00	3.15	4.22
	200	1544.00	711.00	3040.55	1861.28	3.00	4.00	3.00	3.97
	500	2442.50	726.00	4509.15	2094.63	2.50	4.00	2.85	4.46
goana	50	2966.00	655.50	4182.35	2089.35	3.00	4.00	2.55	4.17
	100	1491.00	898.00	3215.40	2102.89	3.00	4.00	3.45	3.96
	200	2823.50	856.00	4270.35	2003.36	2.50	3.50	2.80	3.94
	500	3967.00	720.00	4861.75	2006.60	3.00	4.00	2.95	4.49
topGO	50	394.50	441.50	437.10	793.67	5.00	5.00	5.30	5.27
	100	80.50	151.50	270.75	318.02	7.00	5.00	7.10	5.76
	200	86.00	129.50	251.65	235.31	7.00	6.00	7.20	6.27
	500	60.50	24.50	101.65	111.66	6.00	6.00	6.65	5.95

Remarkably, DAVID, Enrichr, clusterProfiler, and topGO displayed the smallest annotation sizes and the highest depths across all list sizes in both datasets (Tables 2, 3), indicating that these tools tend to obtain more specific enriched ontologies and provide more descriptive results. For example, their results with the 500-hallmark list include, among the top-ranking enriched GO terms, “nucleotide excision repair” (DAVID and Enrichr), “purine ribonucleotide catabolic process” (clusterProfiler), and “canonical glycolysis” (topGO), which are more informative compared to the broader ontologies “primary metabolic process” and “response to stimulus” (Supplementary Table S2).

Moreover, ClueGO’s output contained highly informative ontologies, especially for smaller list sizes (200, 100, and 50 for the Contextual dataset; 50 and 100 for the *Hallmark* dataset), but failed to do so with larger input lists. In this sense, as we tested the 500 lists, generic terms such as “metabolic process” and “biosynthetic process” became much more prevalent.

Conversely, g:Profiler and goana produced substantially less informative results, as they retrieved the largest and shallowest GO terms, which tend to be less precise in terms of biological significance. For instance, goana obtained the ontologies “regulation of biological process” and “metabolic process”, among multiple other generic descriptions, across all results with the *Contextual* and *Hallmark* lists, respectively. On the other hand, g:Profiler yielded more informative terms than goana, but still included rather broad GO terms, especially with the *Contextual* dataset, such as “response to stimulus” and “biological regulation”. However, we observed that both tools obtained smaller, deeper ontologies with the 200- and 100-lists in the *Hallmark* dataset, particularly in the top 20, compared to the top 100.

The remaining tools BiNGO, GOstats, PANTHER, ShinyGO, and WebGestalt provided intermediate results in terms of biological specificity with the Contextual lists. Nonetheless, a pattern similar to g:Profiler and goana with the *Hallmark* lists of sizes 200 and 100 can be observed. For all of these tools, these particular inputs appear to yield more biologically informative results and place them among the highest-ranked terms. Given that the 500 *Hallmark* list is relatively more heterogeneous, as it includes genes from three distinct biological hallmarks (DNA repair, Hypoxia, and Response to UV radiation), while the smaller lists are comprised solely of genes from the hypoxia hallmark, our results suggest that these tools tend to lose specificity when analyzing more heterogeneous gene lists. Furthermore, loss in specificity observed with the 50 *Hallmark* list – which also comprises only hypoxia-related genes – is likely related to the small size of the input. This reinforces our approach by showing that the nature and size of the input data will strongly affect the quality of the FEA results.

**FIGURE 4:**
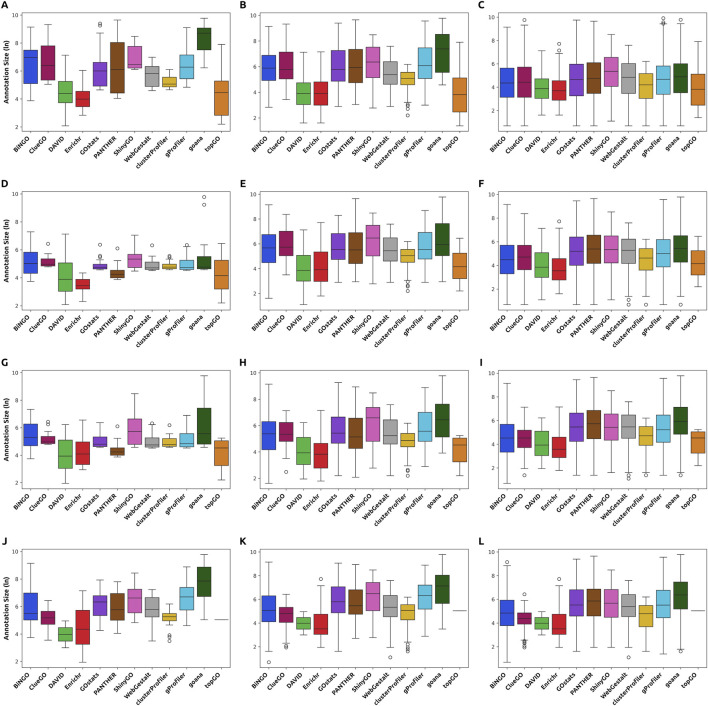
Distribution of the annotation sizes of the enriched ontologies for each *Hallmark* dataset input in the top 20 (first column), top 100 (second column), and all results (third column). Tools display varying distributions of annotation sizes, with some exhibiting a preference for smaller GO terms. The differences are more prominent within the top-ranking ontologies. **(A–C)** Distribution for the 500 Hallmark list in the top 20, 100, and all results, respectively. **(D–F)** Distribution for the 200 *Hallmark* list in the top 20, 100, and all results, respectively. **(G–I)** Distribution for the 100 *Hallmark* list in the top 20, 100, and all results, respectively. **(J–L)** Distribution for the 200 *Hallmark* list in the top 20, 100, and all results, respectively.

**FIGURE 5:**
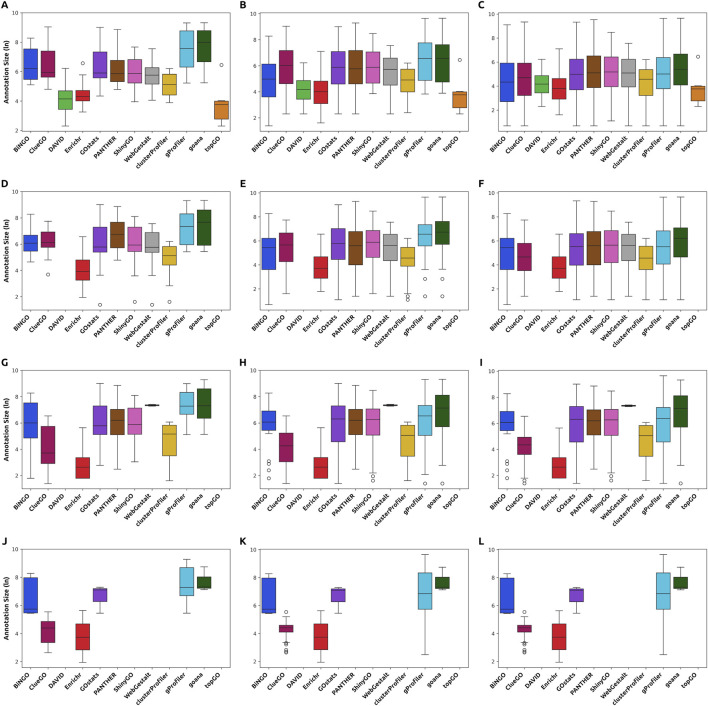
Distribution of the annotation sizes of the enriched ontologies for each *Contextual* dataset input in the top 20 (first column), top 100 (second column), and all results (third column). Tools display varying distributions of annotation sizes, with some exhibiting a preference for smaller GO terms. The differences are more prominent within the top-ranking ontologies. **(A–C)** Distribution for the 500 *Contextual* list in the top 20, 100, and all results, respectively. **(D–F)** Distribution for the 200 *Contextual* list in the top 20, 100, and all results, respectively. **(G–I)** Distribution for the 100 *Contextual* list in the top 20, 100, and all results, respectively. **(J–L)** Distribution for the 200 *Contextual* list in the top 20, 100, and all results, respectively.

Furthermore, by analyzing the annotation size distributions for the top 20, top 100, and all enriched GO terms from each tool, we found that differences in distribution patterns are much more pronounced in the top 20 and top 100 than across all results. Specifically, DAVID, Enrichr, clusterProfiler, and topGO consistently identify smaller ontologies among their top results, suggesting these tools favor more specific GO terms at the top ranks. In contrast, others spread such precise ontologies throughout their results (Figures 4, 5). However, it is essential to note that the output size can influence the comparisons with the full extent of the results, especially in tools that yield larger results. The distribution of GO terms’ annotation size is skewed, with most ontologies having fewer genes annotated to them (Jelier et al., 2008). Consequently, as output size increases, the distribution of annotation sizes in FEA results typically shifts toward smaller values. Also, in the case of ShinyGO, which only outputs up to 1,000 enriched GO terms, distributions can be affected, as not all of the enriched ontologies are accounted for.

The findings above reflect a known issue in the field, widely discussed among users, regarding the relevance of the retrieved GO terms. Users are familiar with the challenge of finding relevant or more descriptive GO terms amidst the vast number of bioprocesses that tools might yield. It is unfeasible or impractical to manually analyze, for instance, more than 5000 GO terms (Figure 2) to identify relevant bioprocesses. Our results indicate that tools differ significantly in the GO terms they rank first and in their level of informativeness.

### 3.4 The degree of stringency differs among tools

We also assessed the tools’ performance in terms of accuracy and FPR by using Wang’s SS to construct a confusion matrix for each FEA result with the *GOBP* dataset. DAVID, WebGestalt, and topGO were the tools that exhibited the highest accuracy values (Table 4). Since accuracy, in the context of this study and of how the confusion matrix was defined, fundamentally denotes how similar and dissimilar the enriched and not enriched GO terms are to the targets for each list, this indicates that the FEA results produced by the selected tools are highly consistent with the biological context of the input list.

**TABLE 4:** Tools performance metrics for all list sizes in the GOBP dataset.

Tool	Size	Accuracy	FPR	Median target rank	Identified targets
BiNGO	500	0.43	0.59	220	10/15
	200	0.44	0.57	91.5	4/7
	100	0.76	0.25	5	3/4
	50	0.41	0.59	4	2/3
ClueGO	500	0.36	0.66	155	15/15
	200	0.28	0.74	28	7/7
	100	0.16	0.86	3	4/4
	50	0.04	1.00	6	3/3
DAVID	500	0.77	0.22	22	14/15
	200	0.81	0.18	17	7/7
	100	0.87	0.12	2.5	4/4
	50	0.87	0.13	9	2/3
Enrichr	500	0.53	0.49	24	13/15
	200	0.50	0.51	8	7/7
	100	0.67	0.34	3	4/4
	50	0.48	0.52	1.5	2/3
GOstats	500	0.40	0.62	134	15/15
	200	0.43	0.58	28	7/7
	100	0.64	0.37	4	4/4
	50	0.44	0.57	6	3/3
PANTHER	500	0.45	0.57	163	15/15
	200	0.49	0.52	37	7/7
	100	0.78	0.22	3.5	4/4
	50	0.54	0.46	28.5	2/3
ShinyGO	500	0.12	1.00	165	15/15
	200	0.05	1.00	51	7/7
	100	0.19	0.82	4	4/4
	50	0.02	1.00	4	3/3
WebGestalt	500	0.63	0.38	102	15/15
	200	0.70	0.29	22	7/7
	100	0.89	0.11	4.5	4/4
	50	0.77	0.23	7	3/3
clusterProfiler	500	0.40	0.62	51	15/15
	200	0.46	0.55	13	7/7
	100	0.69	0.31	3	4/4
	50	0.41	0.60	6	3/3
gProfiler	500	0.33	0.70	168	15/15
	200	0.36	0.66	43	7/7
	100	0.52	0.48	4	4/4
	50	0.38	0.63	6	3/3
goana	500	0.34	0.69	232	15/15
	200	0.41	0.60	59	7/7
	100	0.61	0.39	4	4/4
	50	0.45	0.56	6	3/3
topGO	500	0.79	0.19	17.5	10/15
	200	0.79	0.21	9	6/7
	100	0.74	0.24	1	1/4
	50	0.76	0.24	12	3/3

Additionally, these tools displayed the lowest FPR values, indicating that the GO terms they enrich are highly related to the input data. ClueGO and ShinyGO were the least accurate software in this analysis (Table 4), primarily due to the generation of numerous false positives (i.e., enriched ontologies unrelated to the context of the input list) (Table 4). Moreover, both tools underperformed in terms of FPR due to the nature of their results, which contained few or none non-statistically significant GO terms, thereby biasing FPR towards 1. This result highlights that performance metrics are highly dependent on the method used to define them and the specificities of the tools. Therefore, despite being undoubtedly relevant performance measures, solely relying on such metrics to compare different tools can be problematic in the case of FEA.

**FIGURE 6:**
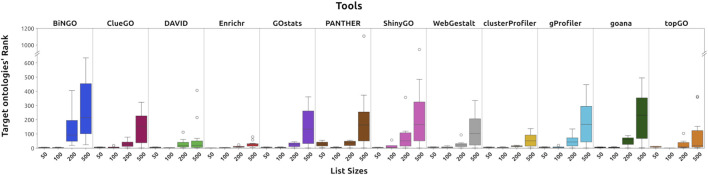
Ranks of the target ontologies for each list in the GOBP dataset. The results show that tools tend to spread the targets throughout the results, especially with bigger inputs. DAVID, Enricher, clusterProfiler, and topGO were less likely to disperse them.

To complement the confusion matrix analysis, we evaluated the identification and ranking of the target ontologies for each list size in the *GOBP* dataset. Regarding identification abilities, most tools were able to enrich 90% or more of the proposed target (Table 4), with the only exceptions being BiNGO and topGO, which failed to do so (66% and 69% identification rates, respectively). In terms of ranking capabilities, across the 100 and 50 lists, all software ranked the identified target GO terms near the top (Figure 6; Table 4). However, most tools lost this ability and increased the dispersion of the targets in the ranking. DAVID, Enrichr, clusterProfiler, and topGO were the only tools that could consistently position the targets within the top-ranking ontologies across all list sizes (Figure 6), suggesting that they tend to prioritize results closely related to the input.

### 3.5 Biological profiles are coherent across tools, but vary in terms of priorization

We further applied MCL to cluster the enriched ontologies for each FEA result with the *Hallmark* and *Contextual* datasets. Headers were manually assigned to the 15 largest clusters to compare the biological information provided by each tool. Notably, with the *Hallmark* lists, most tools identified clusters directly related to the expected biological processes (Supplementary Table S3), namely, DNA repair, hypoxia, and response to UV radiation, since the inputs comprised their respective hallmark genes. The only exceptions were DAVID for the 50-lists and topGO for both 100 and 50-lists, which did not exhibit hypoxia clusters. Both tools did, in fact, enrich at least one hypoxia-related ontology; that enrichment, however, did not cluster with any other enriched GO terms.

As most tools yielded results that were at least partially expected in the *Hallmark* dataset, the most significant differences ultimately come down to the interpretability of their FEA results. Remarkably, DAVID and topGO, two of the tools that had previously produced the most informative results, also displayed the most coherent and precise clusters of GO terms. This observation aligns with the previously reported high accuracy values. In contrast, tools that generated larger and less specific outputs – for instance, BiNGO, ClueGO, GOstats, PANTHER, ShinyGO, WebGestalt, g:Profiler, and goana – enriched a greater number of terms that were weakly aligned with the biological context of the inputs. Clusters that pertained to “nucleotide metabolism”, “organ and system development”, and “regulation of cell fate and metabolism” were the most frequently produced groupings across all lists in this dataset. Although these are somewhat related to the expected biological processes, their GO terms are not as strongly associated as terms such as “DNA repair”, “response to oxygen levels”, or “response to radiation”. The presence of large numbers of these closely related yet unspecific ontologies might, depending on the tools’ ranking abilities, conceal the most insightful or expected descriptions, thus hindering the interpretability of the results.

Moreover, the behaviors observed in the clustering results for the *Contextual* dataset FEA analyses were consistent with those observed in the *Hallmark* dataset (Supplementary Table S4). DAVID still exhibited the most coherent and well-defined clusters for the 500-list, but did not display clustering results for the smaller lists, as it enriched none or only a few GO terms. Similarly, topGO also did not exhibit any clusters for any of the inputs in this dataset for the same reason as DAVID. Nonetheless, in general, the profiles of the biological groups yielded, and, thereby, the overall biological information contained in the enrichment results, were coherent across all tools.

As a consequence, this suggests that the main differences in the biological information contained within the FEA results across all tools lie mainly in their ability to prioritize what’s most relevant in the ranking and to enrich more specific GO terms at the top of the results.

## 4 Discussion

FEA has become a crucial step in studies that rely on omics data to drive biological discovery, as it provides a facilitated way to interpret such information. Given its valuable role, several performance studies have been conducted to compare the various tools that employ FEA (Tarca et al., 2013; Bayerlová et al., 2015; Lim et al., 2018; Nguyen et al., 2019; Zyla et al., 2019; Geistlinger et al., 2021; Buzzao et al., 2024). Nevertheless, such studies overlook the biological informativeness provided by FEA while focusing comparisons on metrics that, although indispensable, fail to accurately describe the performance of FEA tools. As the final step of FEA inevitably involves manual interpretation by the user, it is of utmost importance, in the context of FEA, to also assess the tools’ biological precision and interpretability.

In this study, we addressed the aforementioned limitation by developing a novel benchmarking strategy centered around the biological significance of the results. We also provide an extensive analysis of 12 widely used ORA-based GO FEA tools.

By exploiting the GO structure and assessing the biological specificity of the enriched GO terms through their annotation size and depth, we identified insightful tendencies commonly overlooked in other studies. Our results show that DAVID, Enrichr, clusterProfiler, and topGO yield more informative results than the other tools, especially compared with goana and g:Profiler, which tend to enrich more generic ontologies. It is essential to note that alternative methods exist for evaluating the specificity of a GO term. For instance, Information Content (IC), which is essentially calculated based on the annotation size of an ontology and its offspring, and the number of offspring GO terms, are two viable options (Louie et al., 2010; Tomczak et al., 2018). However, the measures employed here, annotation size and depth, are much more straightforward to grasp for the average FEA user, as they are direct properties of the GO and don’t require additional techniques to evaluate them.

Nonetheless, there is currently no gold-standard method for assessing GO term biological specificity, and these metrics display inherent limitations. For instance, the annotation size is directly affected by annotation coverage, which is particularly problematic when dealing with non-model organisms. Additionally, two terms being at the same depth in the ontology does not necessarily imply that both descriptions are equally specific. Despite these limitations, leveraging the GO’s properties (i.e., term annotation size and depth) to estimate specificity remains an intuitive and valid strategy (Lewin and Grieve, 2006; Louie et al., 2010; Tomczak et al., 2018). Regardless of the chosen method, we advocate incorporating metrics that reflect the informativeness of results in FEA benchmark studies, such as those described above.

Additionally, our results suggest that the interpretability of each tool’s results may be a key factor in the differences between the selected tools. In this regard, we argue that output sizes and ranking abilities are direct measures of the intelligibility of the biological context provided by FEA. Large results can be challenging to interpret, and the most insightful GO terms may be overlooked amid the ocean of enriched ontologies. Likewise, the ability to position the most relevant terms at the top of the results is a quality instrumental in the case of FEA. Tools with such tendencies may benefit from the usage of additional techniques that make their results more interpretable. Redundancy reduction strategies have been developed to facilitate the interpretation of FEA results (Jantzen et al., 2011; Ozisik et al., 2022); however, it is crucial to ensure that such approaches don’t remove the most specific descriptions while retaining the less informative generic terms. For example, using subsets of the GO that contain no redundant terms, such as the GO slim, may decrease redundancy in FEA results but at the cost of informativeness, as these downsized subsets tend to span broader ontologies. Besides, methods that group the enriched GO terms into functional clusters, as we did in this study using MCL, can be compelling for uncovering the biological profile of the results, further increasing interpretability, especially since functionally related ontologies might be scattered across the full extent of the results.

Furthermore, resources that implement GO term clustering commonly rely on the GO structure or semantic similarity to determine functionally related clusters (Klopfenstein et al., 2018; Xin et al., 2022; Xu et al., 2024). Nonetheless, most platforms of this kind are optimized to work only with their own imbued FEA outputs. Tools capable of universally conducting such interpretability-oriented techniques to FEA outputs – regardless of the tools that produced them – are still needed in the field.

We also tested the tools with four different input sizes to investigate their impact on the generated GO profile. The analyses we conducted revealed that input size primarily affects output sizes and the tools’ ranking abilities by dispersing the expected targets for larger sizes. Tools are affected differently: DAVID, Enrichr, clusterProfiler, and topGO, which, curiously, were also the ones to provide more biologically specific results, prioritized the targets by placing them closer to the top of the results, and were less prone to dispersing them. This suggests that these tools tend to position the GO terms strongly related to the input among the top-ranking categories, thereby displaying results that are easier for the user to interpret. For the user, this is relevant because it helps select the most appropriate tool based on the research question. In this sense, hypothesis-oriented studies may take advantage of tools that yield larger, less stringent results, while studies aiming to confirm a biological response will mostly benefit from more conservative tools. Likewise, our results might aid researchers in selecting tools that better fit their input size, as different software exhibit heterogeneous ranking and GO identification performance across input sizes.

The results presented here demonstrate that, even when using the same FEA method, different tools can yield substantially different results. These discrepancies are primarily attributable to (i) variations in GO and annotation versions, (ii) modifications within the statistical approaches themselves, and (iii) the unique algorithms each tool employs for statistical testing. The GO database is regularly updated, with terms being added or removed and annotation sources being revised. Such updates can significantly affect FEA results and change their biological interpretation (Tomczak et al., 2018). Additionally, the implementation of the ORA method and the statistical correction can vary slightly across tools. For example, DAVID uses a modified version of Fisher’s Exact Test, known as the EASE score, which subtracts 01 from the number of input genes mapped to a given pathway, thereby conferring more conservative performance than the original Fisher’s Exact Test. Similarly, differences in how statistical correction methods are implemented can also influence results and their biological interpretation (Ziemann et al., 2024). Finally, the algorithms used to perform multiple statistical tests and generate outputs can produce significant differences in results across tools. For instance, the default algorithm in topGO implements a technique that essentially filters out redundant terms while retaining the ontologies that best characterize the input list, thereby preserving its biological relevance. Likewise, g:Profiler, WebGestalt, clusterProfiler, and ClueGO include built-in options that perform similar functions, but they were outside the scope of this study, as they are optional post-processing steps rather than core components of the algorithms. Users should be mindful of the selected tool’s specific features to ensure they obtain the most meaningful results from FEA.

Ultimately, the choice of the appropriate tool is determined primarily by the user’s analysis objectives and the nature of the input data. For analyses aimed at investigating novel biological pathways that have not been previously described within a given condition, tools that yield a larger number of enriched terms and more specific terms, such as clusterProfiler and Enrichr, are preferable. Furthermore, these tools and others that retrieve broader terms (i.e., BiNGO, ClueGO, GOstats, PANTHER, WebGestalt, and g:Profiler) are also suitable for highly heterogeneous data, as more stringent software may exclude loosely related yet potentially interesting descriptions. Conversely, more conservative approaches, exemplified by tools like DAVID and topGO, may be more suitable when the primary objective is to validate existing hypotheses and the input data is coherent, such as when the biological condition in question is known to be associated with specific bioprocesses or when the study focuses mainly on genes known to participate in overlapping pathways. Moreover, the user should be aware of the tools’ characteristics and outputs when selecting software and performing FEA. For instance, tools that yield larger and broader results may require filtering and visualization strategies to facilitate interpretation. Many of the tools selected in this study include such features embedded in the software. For example, ClueGO, clusterProfiler, and DAVID provide functional clustering options. Similarly, ClueGO, ShinyGO, WebGestalt, clusterProfiler, and g:Profiler all provide filtering strategies (e.g., redundancy removal functions, GO term size filtering, and GO term depth filtering) and built-in visualizations. Such features significantly increase the usability of FEA tools. Likewise, users should consider tools’ characteristics that are not directly related to the FEA itself. As mentioned earlier, FEA tools offer a wide range of additional features that can significantly impact analysis efficiency; consequently, the quality of documentation and tutorials plays a central role in FEA tool selection. Exceptionally, all the tools included in our study have satisfactory documentation on practical usability. However, some of them do not report key information, for example, the exact GO and annotation versions being used to conduct FEA, and that is quite problematic in the FEA field, especially because it hinders reproducibility.

Taken together, our results highlight aspects of the FEA tool’s behavior that are typically overlooked and provide relevant information that better guides the selection of FEA software.

## Data Availability

The input datasets and scripts can be acquired from https://github.com/LARA-Lab-Aging.
