# Supplementary material for: Benchmarking multiple gene ontology enrichment tools reveals high biological significance, ranking, and stringency heterogeneity among datasets
Source: Front Bioinform. 2026 Jan 29;6:1755664. doi: 10.3389/fbinf.2026.1755664 (PMC12894344; doi:10.3389/fbinf.2026.1755664)
Supplement: Supplementary file 6 [file Image1.pdf]

Supplementary Figures

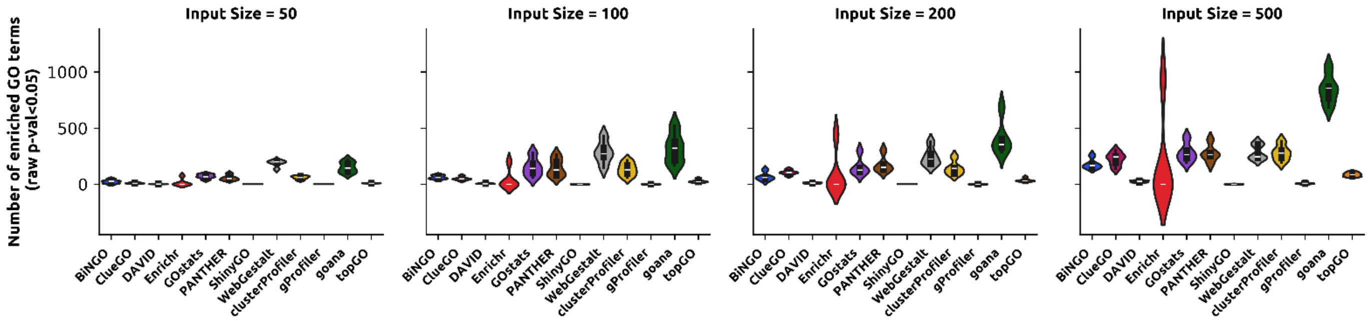

Supplementary Figure 1. Distribution of the number of enriched GO terms for the input lists in the *Random* dataset. ClueGO and goana tend to yield enrichment results for data with no biological context (i.e., false positives).
